# Long-Term Effects of Mobile-Based Metamemory Cognitive Training in Older Adults With Mild Cognitive Impairment: 15-Month Prospective Single-Arm Longitudinal Study

**DOI:** 10.2196/81648

**Published:** 2026-01-02

**Authors:** Jung-In Lim, Yeeun Byeon, Sunyoung Kang, Hyeonjin Kim, Keun You Kim, Lukas Stenzel, So Yeon Jeon, Jun-Young Lee

**Affiliations:** 1 Interdisciplinary Program in Cognitive Science Seoul National University Seoul Republic of Korea; 2 Department of Medical Device Development Seoul National University College of Medicine Seoul Republic of Korea; 3 Department of Psychiatry Institute of Behavioral Science in Medicine Yonsei University College of Medicine Seoul Republic of Korea; 4 Department of Psychiatry Seoul Metropolitan Government-Seoul National University Boramae Medical Center Seoul Republic of Korea; 5 Cogthera GmbH Munich Germany; 6 Department of Psychiatry Seoul National University College of Medicine Seoul Republic of Korea

**Keywords:** Alzheimer disease, cognitive training, digital technology, mild cognitive impairment, metamemory

## Abstract

**Background:**

Mild cognitive impairment (MCI) is an intermediate state between normal aging and dementia, characterized by subjective cognitive decline and objective memory impairment. Cognitive training has consistently shown short-term benefits for individuals with MCI, but evidence on the long-term effectiveness is extremely limited. Given the progressive nature of MCI and the need for sustainable strategies to delay cognitive decline, research on the long-term impact of cognitive training is necessary and timely. Mobile-based platforms offer a promising solution by enhancing accessibility and adherence, but their durability of effect over extended periods remains underexplored.

**Objective:**

This study aimed to evaluate the long-term effects of a mobile-based cognitive training app on the cognitive function of older adults with MCI.

**Methods:**

In total, 28 older adults with MCI used Cogthera, a mobile cognitive training app based on metamemory training. Participants completed 2 training sessions daily for 3 months, and 9 (32%) continued for an additional 12 months. Cognitive function and quality of life were assessed using the Alzheimer’s Disease Assessment Scale-Cognitive Subscale 14 and EQ-5D-5L.

**Results:**

Cognitive function improved over 15 months, as measured by Alzheimer’s Disease Assessment Scale-Cognitive Subscale (*F*_2,35.56_=7.08; *P*=.003). EQ-5D-5L scores increased at 3 months but did not show sustained change at 15 months (*F*_2,42.14_=3.40; *P*=.04). Greater cognitive improvements were associated with younger age, higher functional status, and lower baseline cognitive function.

**Conclusions:**

This study showed that long-term use of a mobile-based metamemory cognitive training app was associated with cognitive improvements over 15 months. Although limited by the small sample size and the absence of a control group, these findings suggest potential for mobile cognitive training as a sustainable intervention that warrants validation in larger trials.

## Introduction

### Background

Mild cognitive impairment (MCI) is an intermediate stage between normal aging and Alzheimer disease (AD). It has emerged as a key target for early intervention to delay AD. MCI is characterized by subjective cognitive decline along with objective memory impairment without functional impairment [1]. The prevalence of MCI is 6.7% among individuals aged between 60 and 64 years and 25.5% among those aged between 80 and 84 years [2]. In addition, approximately 10% of individuals with MCI progress to AD annually [3].

Nonpharmacological interventions, such as physical activity, social engagement, and cognitive training, are widely recommended to delay AD onset in MCI [4-7]. Cognitive training has demonstrated superior efficacy in improving cognitive function in short-term treatment [8-11]. Despite these short-term benefits, few studies have examined whether cognitive training produces sustained effects beyond 1 year because of methodological difficulties [12]. Traditional cognitive interventions also rely on in-person, paper-based sessions, which can limit accessibility and adherence [13]. Digital cognitive training based on a mobile app enables home-based, self-paced engagement and has demonstrated greater participation [14].

We have previously reported the short-term efficacy of metamemory training (MMT) [15,16]. Metamemory refers to an individual’s awareness and understanding of their memory functions, including their contents and processes [17,18]. MMT is based on this concept and has been shown to be effective in teaching mnemonic strategies and improving cognitive function [19-22]. However, its long-term efficacy may need to be investigated, especially with a mobile cognitive training app.

### Objectives

This study aimed to examine the long-term effects of a cognitive training app based on MMT in older adults with MCI. We hypothesized that prolonged use of the app would lead to sustained improvements in cognitive function.

## Methods

### Participants

Participants were recruited from 1 memory clinic. Inclusion criteria were as follows: (1) aged between 55 and 85 years; (2) diagnosed with MCI by trained psychiatrists or neurologists according to the criteria proposed by Petersen [23]; (3) ownership of a personal smartphone and no difficulties using mobile apps; (4) if taking cognitive enhancers (acetylcholinesterase inhibitors or memantine), a stable dose maintained for at least 12 weeks before randomization; (5) availability of a caregiver who spends more than 8 hours per week with the participant and agrees to assist with follow-up and clinical evaluations; (6) ability to independently make phone calls to a caregiver using a smartphone; (7) no difficulties with reading or writing in Korean; and (8) adequate vision and hearing to participate in the clinical trial. MCI diagnosis was based on the following criteria by Petersen [23]: (1) reported concerns regarding cognitive changes, (2) impairment in 1 or more cognitive domains, (3) preservation of independence in functional abilities, and (4) absence of dementia [24]. Participants underwent cognitive assessments conducted by clinical psychologists using the Clinical Dementia Rating (CDR) and the Korean version of the Consortium to Establish a Registry for Alzheimer’s Disease Neuropsychological Assessment Battery (CERAD-NP). Eligibility criteria required a global CDR score of 0.5 and performance at least 1.0 SD below the mean in 1 or more memory domains of the CERAD-NP.

Exclusion criteria included the presence of serious physical illness or psychiatric disorders that could interfere with study procedures; the use of medications known to affect cognitive function (except those taken consistently for at least 3 months); and any neurological or medical conditions associated with cognitive decline other than MCI, such as stroke, central nervous system infection, head trauma, alcohol dependence, or depression. Of the 40 participants screened, 10 (25%) were excluded, 1 (3%) dropped out, and 1 (3%) experienced a stroke. Data from 28 (70%) participants were analyzed.

### Ethical Considerations

This study was reviewed and approved by the institutional review board of Seoul Metropolitan Government-Seoul National University Boramae Medical Center (20-2022-48). All participants provided informed consent before participation. All collected data were deidentified and stored securely, and no personally identifiable information was accessible to the research team. Participants received KRW 200,000 (US $136) as compensation for their participation.

### Intervention

Participants underwent cognitive training using Cogthera, a smartphone-based app based on MMT developed by Youn et al [16]. The structure and content of the Cogthera intervention, including its implementation of metamemory-based strategies, have been previously described in detail. The program was designed to enhance key cognitive functions, including attention, imagery, and association, which support effective memory encoding and retrieval. Attention training helped participants focus and concentrate on target information to facilitate deeper encoding. Imagery training encouraged vivid visualization to reinforce memory consolidation and retrieval. Association training promoted the integration of new information into existing semantic memory networks. Throughout the training process, Cogthera provides personalized feedback and dynamically adjusts task difficulty, enabling users to observe and evaluate their cognitive processes independently.

A 15-month single-arm longitudinal study was conducted to investigate the long-term efficacy of mobile-based cognitive training. Participants completed a 3-month cognitive training program using Cogthera, with 9 (32%) of the 28 participants continuing an additional 12 months. The training program consisted of 2 daily sessions, 7 days per week, with each session lasting approximately 15 minutes. The first session comprised 3 core cognitive exercises targeting attention, imagery, and association. The second session included 4 additional exercises, selected from 9 available options, excluding those already used in the first session. A personalized algorithm determined daily exercise composition to optimize engagement. All participants were able to use the Cogthera program independently without external assistance. The detailed content of each exercise can be found in Multimedia Appendix 1.

### Measures

#### Alzheimer’s Disease Assessment Scale-Cognitive Subscale 14

The Alzheimer’s Disease Assessment Scale-Cognitive Subscale 14 (ADAS-Cog 14) was used to assess the severity of cognitive dysfunction [25]. This measure consists of fourteen tasks: (1) word recall, (2) commands, (3) constructional praxis, (4) delayed word recall, (5) naming, (6) ideational praxis, (7) orientation, (8) word recognition, (9) maze, (10) number cancelation, (11) remembering instructions, (12) comprehension, (13) word finding difficulty, and (14) spoken language ability. The ADAS-Cog 14 subdomains were categorized into 3 cognitive domains: memory, language, and praxis [26]. The total ADAS-Cog 14 score ranges from 0 to 90 points, with higher scores indicating greater cognitive impairment, as the score reflects the number of errors made across tasks.

#### CERAD-NP Assessment

The CERAD-NP was administered to assess cognitive function [27,28]. This battery included the following neuropsychological tests: (1) verbal fluency, (2) Boston Naming Test, (3) Mini-Mental State Examination, (4) word list learning, (5) constructional praxis, (6) word list recall, (7) word list recognition, (8) constructional praxis recall, (9) Trail Making Test, and (10) Stroop test. The CERAD-NP total score was calculated as the sum of raw scores, with higher scores indicating better cognitive function.

#### CDR Scale

The CDR scale was used to stage dementia severity [29]. This scale is informed by semistructured interviews conducted with both participants and informants, covering 6 domains: memory, orientation, judgment and problem-solving, community affairs, home and hobbies, and personal care. Each domain is scored from 0 to 3, with the scores used in calculating the global CDR score and the CDR-Sum of Boxes (CDR-SB). The global CDR score is an ordinal scale ranked from 0 to 3 as follows: 0=no cognitive impairment, 0.5=questionable or MCI, 1=mild dementia, 2=moderate dementia, 3=severe dementia. The CDR-SB is a continuous measure ranging from 0 to 18, calculated by summing the individual domain scores. Higher scores on both the global CDR and CDR-SB indicate greater cognitive and functional impairment.

#### EQ-5D-5L Scale

The EQ-5D-5L was administered to evaluate health-related quality of life [30]. This measure consists of five dimensions: (1) mobility, (2) self-care, (3) usual activities, (4) pain and discomfort, and (5) anxiety and depression. Each dimension is rated on a 5-level scale, with response options ranging from *no problem* to *extreme problem*. Responses were converted into a single index score using a national-specific value set [31]. This value set, derived from stated preference data collected from the general population, assigns weights to each health dimension level. The index score ranges from 0 to 1, with higher scores indicating better overall health status. In contrast, a lower score on each subdimension reflects a higher level of health in that specific domain.

### Data Collection

Participants who provided informed consent were screened using global CDR and CERAD-NP. During the initial visit, participants underwent cognitive function assessments using ADAS-Cog 14 and completed self-reported questionnaires, such as EQ-5D-5L. After completing these assessments, they were provided with a smartphone preinstalled with Cogthera. Follow-up data on ADAS-Cog 14 and EQ-5D-5L were collected after 3 months of using Cogthera. In addition, further assessments were conducted on 9 (32%) of the 28 participants who continued using Cogthera for 15 months. Training adherence was assessed using compliance, which was defined as the proportion of completed sessions relative to the total number of assigned sessions. The assigned frequency was 2 sessions per day, and compliance was calculated separately for the initial 3-month period and the 12-month extension.

### Statistical Analysis

Linear mixed model analyses were performed to assess changes in ADAS-Cog 14 and EQ-5D-5L scores over time while accounting for both intraindividual and interindividual variations in longitudinal data. The models included time (baseline, 3 mo, and 15 mo) as a fixed factor and participant as a random effect, allowing individual variability in intercepts. To evaluate the overall effect of time, type Ⅲ ANOVA was conducted.

To identify explanatory variables predicting intervention effects, the least absolute shrinkage and selection operator (LASSO) regression was used. This approach was used to identify the most relevant predictors while minimizing overfitting [32]. The outcome variable was the change in ADAS-Cog 14 at follow-up, with LASSO models including age, sex, education, CDR-SB, baseline ADAS-Cog 14 scores, and baseline EQ-5D-5L scores as predictors. All variables were standardized by centering each variable around the mean and scaling by the SD. The regularization parameter, λ, was optimized using 9-fold cross-validation to balance model complexity and predictive performance. The optimal λ value was then applied to estimate the coefficients of the selected predictors.

To contextualize cognitive changes observed in this study, descriptive data from the placebo cohort of EXPEDITION studies were used as a historical reference [33,34]. EXPEDITION and EXPEDITION-2 were multicenter, double-blind, phase 3 trials of solanezumab, including 663 patients with MCI treated with a placebo. Cognitive function was assessed using ADAS-Cog 14 at baseline and at 6 follow-up points every 3 months over 18 months. Welch *t* test (2-tailed) was conducted to compare data from this study with the EXPEDITION placebo cohort at baseline, 3 months, and 15 months, accounting for unequal variances and sample sizes.

All statistical analyses were conducted using R software (version 4.4.1; R Foundation for Statistical Computing). Statistical significance was set at *P*<.05, with all tests being 2-tailed. For the post hoc analysis of the linear mixed model, Bonferroni-adjusted *P* values were reported. Effect sizes for the fixed time effects were also reported, with partial eta-squared (η^2^_p_) computed from the corresponding *F* statistics. Missing data were addressed through complete case analysis without imputation. To ensure analytic independence, all data access, monitoring, and statistical analyses were conducted exclusively by authors not affiliated with the company. Company-affiliated authors had no access to raw data and did not participate in data cleaning, analytic decisions, or interpretation of results.

## Results

### Demographic Characteristics

A total of 28 participants were included in the analysis (Table 1). The mean age of participants was 72.8 (SD 6.7) years, and the mean education level was 11.4 (SD 5.0) years. The sample comprised a higher proportion of female individuals (20/28, 71%) and was predominantly composed of nondrinkers (27/28, 96%) and nonsmokers (26/28, 93%). The mean CDR-SB score was 2.0 (SD 1.1), ranging from 0.5 to 4.0.

**Table 1:** Baseline demographic data for the participants (N=28).

Demographic characteristic		Values
Age (y), mean (SD)		72.8 (6.7)
**Sex, n (%)**		
	Female	20 (71)
	Male	8 (29)
Education (y), mean (SD)		11.4 (5.0)
**Consortium to Establish a Registry for Alzheimer’s Disease Neuropsychological Assessment Battery (*z* score^a^), mean (SD)**		
	Word list learning	–0.5 (0.9)
	Word list recall	–1.6 (1.0)
	Word list recognition	–1.7 (1.4)
	Constructional praxis recall	–1.3 (0.9)
Clinical Dementia Rating-Sum of Boxes (score), mean (SD)		2.0 (1.1)

^a^A standardized score indicating how many SDs a value is from the mean.

### Training Adherence

During the initial 3-month training period, participants completed an average of 85.3% (SD 23.6%) of assigned sessions. Among 9 (32%) of the 28 participants who continued for an additional 12 months, mean compliance during the extension period was 51.7% (SD 25.1%).

### Intervention Effects of Cogthera

Linear mixed model analysis revealed a significant decrease in total ADAS-Cog 14 scores over time, indicating improved cognitive function (*F*_2,35.56_=7.08; *P*=.003; η^2^_p_=0.28; Table 2). Specifically, a significant decrease in total ADAS-Cog 14 scores was observed over 15 months (Bonferroni-adjusted *P*=.001), while the change over 3 months was not significant (Bonferroni-adjusted *P*=.27; Table 3).

**Table 2:** Effects of time on cognitive function and quality of life.

		Baseline (n=28), mean (SD)	3 mo (n=28), mean (SD)	15 mo (n=9), mean (SD)	*F* test (*df*)	*P* value
**Alzheimer’s Disease Assessment Scale-Cognitive Subscale^a^**						
	Total score	28.18 (8.21)	27.07 (7.73)	25.56 (8.28)	7.08 (2, 35.56)	.003
	Memory^b^	22.25 (6.24)	21.86 (6.29)	20.44 (8.56)	2.53 (2, 35.83)	.09
	Language^c^	0.86 (0.93)	0.64 (0.83)	0.56 (0.73)	1.64 (2, 35.83)	.21
	Praxis^d^	2.32 (1.19)	1.96 (1.10)	1.33 (1.00)	7.11 (2, 39.33)	.002
**EQ-5D-5L**						
	Index score	0.81 (0.08)	0.85 (0.04)	0.84 (0.07)	3.40 (2, 42.14)	.04

^a^Lower scores represent better performance.

^b^Sum of word recall, delayed word recall, orientation, and word recognition task scores.

^c^Sum of naming, remembering instructions, comprehension, word finding difficulty, and spoken language ability task scores.

^d^Sum of commands, constructional praxis, and ideational praxis task scores.

**Table 3:** Longitudinal changes in cognitive function and quality of life.

				Estimate (SE)		*t* test (*df*)		*P* value^a^
**Alzheimer’s Disease Assessment Scale-Cognitive Subscale**								
	**Total score**							
		3 months vs baseline	–1.11 (0.72)		–1.53 (35.06)		.27	
		15 months vs baseline	–4.33 (1.16)		–3.74 (35.96)		.001	
	**Memory**							
		3 months vs baseline	–0.39 (0.68)		–0.58 (35.17)		.99	
		15 months vs baseline	–2.42 (1.08)		–2.25 (36.38)		.06	
	**Language**							
		3 months vs baseline	–0.21 (0.12)		–1.81 (34.65)		.16	
		15 months vs baseline	–0.09 (0.19)		–0.47 (36.83)		.99	
	**Praxis**							
		3 months vs baseline	–0.36 (0.23)		–1.53 (35.78)		.27	
		15 months vs baseline	–1.35 (0.36)		–3.76 (42.63)		.001	
**EQ-5D-5L**								
	**Index score**							
		3 months vs baseline	0.04 (0.02)		2.58 (35.29)		.03	
		15 months vs baseline	0.03 (0.02)		1.27 (49.19)		.42	

^a^Bonferroni-adjusted *P* values.

In the ADAS-Cog 14 subdomain analysis, praxis scores, representing executive function, showed a significant decline over time (*F*_2,39.33_=7.11; *P*=.002; η^2^_p_=0.27), particularly over 15 months (Bonferroni-adjusted *P*=.001). The reduction in memory scores was marginal (*F*_2,35.83_=2.53; *P*=.09; η^2^_p_=0.12), with a trend-level decrease observed at 15 months (Bonferroni-adjusted *P*=.06). The overall effect of time on language scores was not significant.

A significant change in the EQ-5D-5L index score was observed over time (*F*_2,42.14_=3.40; *P*=.04; *η*^2^*_p_*=0.14), with a statistically significant increase from baseline to 3 months (Bonferroni-adjusted *P*=.03). Among the EQ-5D-5L subdimensions, mobility showed a marginal overall effect of time (*F*_2, 39.05_=2.59; *P*=.09; η^2^_p_=0.12), with a trend-level decrease observed at 3 months (Bonferroni-adjusted *P*=.06). Detailed results for the EQ-5D-5L subdimensions are provided in Tables S1 and S2 in Multimedia Appendix 2).

### Factors Associated With Intervention Effects on Cognitive Function

According to the LASSO results, CDR-SB and baseline ADAS-Cog 14 scores predicted score changes over 3 months, while age, CDR-SB, and baseline ADAS-Cog 14 scores predicted score changes over 15 months. The multiple linear regression model, which included sex, CDR-SB, and baseline ADAS-Cog 14 scores, explained a significant proportion of the variance in score changes over 3 months (*R*^2^=0.40; adjusted *R*^2^=0.33; *P*=.01; Table 4). Specifically, lower CDR-SB scores (β=–0.51; *P*=.02), and higher baseline ADAS-Cog 14 scores significantly predicted greater improvements (β=0.61; *P*=.01).

**Table 4:** Factors predicting cognitive function improvements.

		Values, estimate (SE)	β	*t* test *(df)*	*R*^2^ (adjusted *R*^2^)		*P* value	
**Change over 3 months^a^**						0.40 (0.33)		.01
	Sex	2.60 (1.32)	0.69	1.97			.06	
	CDR-SB^b^	–1.78 (0.70)	–0.51	–2.53			.02	
	ADAS-Cog 14^c^	0.28 (0.09)	0.61	3.01			.01	
**Change over 15 months^d^**						0.81 (0.70)		.03
	Age	–0.44 (0.14)	–0.62	–3.23			.02	
	CDR-SB	–4.80 (1.39)	–1.10	–3.47			.02	
	ADAS-Cog 14	0.61 (0.14)	1.05	4.25			.01	

^a^Score obtained by subtracting the Alzheimer’s Disease Assessment Scale-Cognitive Subscale score at 3 months from the baseline Alzheimer’s Disease Assessment Scale-Cognitive Subscale score.

^b^CDR-SB: Clinical Dementia Rating-Sum of Boxes.

^c^ADAS-Cog 14: Alzheimer’s Disease Assessment Scale-Cognitive Subscale.

^d^Score obtained by subtracting the Alzheimer’s Disease Assessment Scale-Cognitive Subscale score at 15 months from the baseline Alzheimer’s Disease Assessment Scale-Cognitive Subscale score.

The regression model, which included age, CDR-SB, and baseline ADAS-Cog 14 scores, accounted for a significant proportion of the variance in score changes observed over 15 months (*R*^2^=0.81; adjusted *R*^2^=0.70; *P*=.03). Specifically, younger age (β=–0.62; *P*=.02), lower CDR-SB scores (β=–1.10; *P*=.02), and higher baseline ADAS-Cog 14 scores significantly predicted greater improvements (β=1.05; *P*=.01).

### Comparison With Historical Placebo Data

**Figure 1:**
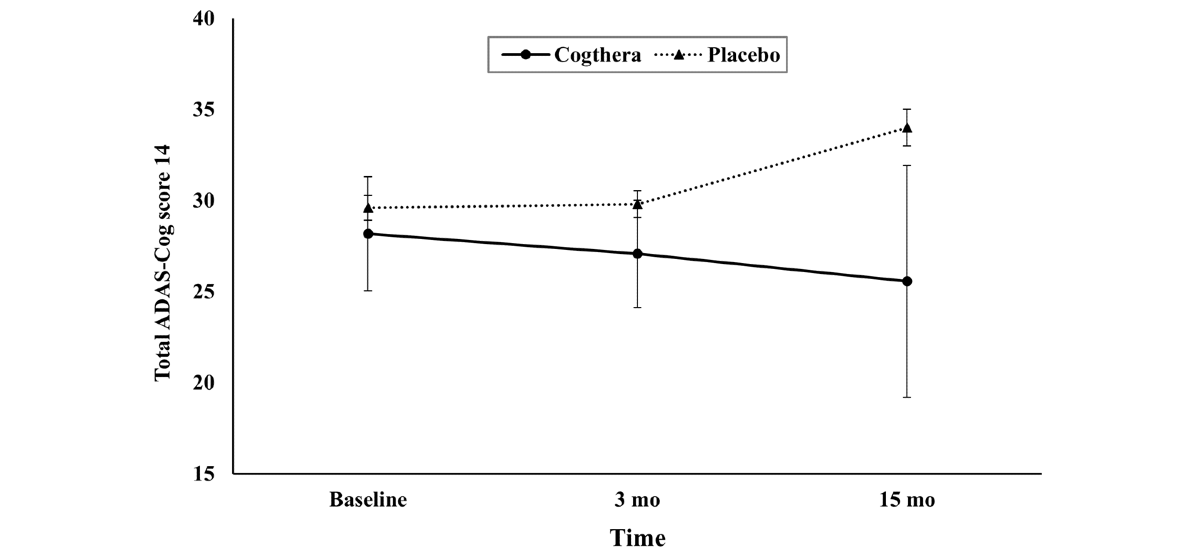
Comparison of cognitive function between the Cogthera group and the historical placebo group. Error bars represent 95% CIs of the mean Alzheimer’s Disease Assessment Scale-Cognitive Subscale 14 (ADAS-Cog 14) scores at each time point.

The mean and SD data from a previously reported placebo cohort were used for comparison at each time point. No significant differences were observed between the Cogthera group and the placebo group at baseline (t_29.68_=0.89; *P*=.38; Figure 1). At 3 months, ADAS-Cog 14 scores in the Cogthera group were marginally lower than those in the placebo group (t_30.63_=1.81; *P*=.08). By 15 months, the difference became statistically significant, suggesting greater cognitive improvement in the Cogthera group (*t*_8.55_=3.01; *P*=.02).

## Discussion

### Principal Findings

This study examined the long-term effects of a mobile-based cognitive training app in older adults with MCI. ADAS-Cog 14 scores decreased over time, and EQ-5D-5L scores increased in the early phase of training. Age, CDR-SB, and baseline ADAS-Cog 14 scores were associated with the degree of cognitive change. Although comparisons with a historical placebo cohort are limited by differences in design and sample characteristics, the observed reduction in ADAS-Cog 14 scores over 15 months suggests a pattern of cognitive change associated with long-term training use.

The primary finding of this study is the reduction in ADAS-Cog 14 total, memory, and praxis scores associated with long-term use of Cogthera. While no meaningful change was observed at 3 months, a clearer reduction emerged at 15 months. This delayed pattern may reflect the cumulative effects of training, but it could also be influenced by practice effects, familiarity with test procedures, or natural fluctuations in cognitive performance. These possibilities underscore the need for cautious interpretation, particularly in the absence of a control group. Even so, the overall pattern is consistent with previous studies, suggesting that longer training durations are more likely to yield detectable cognitive improvements [35]. Unlike other ADAS-Cog 14 subdomains, language scores did not show meaningful change. The small numerical variation observed is likely to reflect measurement variability or stabilization rather than the true effect of the intervention. This aligns with previous research indicating that language tends to remain relatively preserved in the early stage of cognitive decline compared with other ADAS-Cog 14 subdomains [36,37].

Another finding is a rise in the EQ-5D-5L index score during the first 3 months of training. This early change is consistent with previous studies reporting short-term benefits in quality of life among individuals with MCI, but the effect did not persist at 15 months in this study [38-41]. This pattern suggests that changes in perceived well-being may be limited to the initial phase of training. Such early gains may also reflect nonspecific factors, including placebo effects or heightened engagement at the beginning of training.

Predictor analyses revealed that both short- and long-term improvements in cognitive function were associated with lower baseline cognitive function and higher functional status. Participants with higher baseline ADAS-Cog 14 scores, which indicate poorer initial cognitive performance, and those with lower CDR-SB scores, which indicate less functional impairment, tended to show larger reductions over time. Previous research has reported conflicting findings regarding the relationship between baseline cognitive performance and cognitive training effectiveness [42]. Two competing hypotheses have been proposed to explain this relationship: the compensation effect and the magnification effect. The compensation effect suggests that individuals with higher baseline cognitive performance benefit less from training due to limited room for improvement. In contrast, the magnification effect proposes that they benefit more by leveraging greater cognitive efficiency and plasticity. The findings of this study align with the compensation effect, as participants with lower baseline cognitive performance showed greater training-related gains. This pattern suggests that Cogthera may have provided compensatory cognitive stimulation. Such stimulation could have helped individuals with lower baseline cognitive performance make better use of their remaining cognitive resources. CDR-SB reflects the pathological progression of cognitive and functional decline and reliably differentiates MCI from very early AD while predicting future progression [43-45]. The association between lower CDR-SB scores and greater cognitive gains may suggest that individuals with preserved daily function but greater cognitive vulnerability were more responsive to training. These observations are broadly consistent with compensatory mechanisms, while other explanations remain possible.

Younger age was associated with modestly better cognitive outcomes over the 15-month period. One possible explanation is that younger participants may engage more consistently in long-term digital interventions. Previous research has shown that they tend to complete more training sessions and demonstrate higher adherence. Such patterns of adherence may help account for the cognitive changes observed in this group [46-48].

These findings suggest that MMT delivered through a mobile app may offer a scalable and accessible approach for individuals with MCI. Mobile apps provide a practical alternative to traditional cognitive training by allowing users to engage in cognitive exercises at their convenience. As memory decline has already begun in individuals with MCI, this approach may serve as a supportive digital complement to existing strategies. A potential strength of this study is the observation that cognitive changes became more evident with prolonged use, which may help inform future work on long-term digital training. Given the lack of known adverse effects, mobile-based cognitive training could represent a feasible and well-tolerated adjunctive approach for older adults with MCI. It may be particularly useful in cases where pharmacological treatments carry a higher risk of side effects.

### Limitations

Despite the observational insights provided by this study, several limitations warrant careful consideration. First, the absence of a control group limits internal validity, as it is not possible to distinguish training-related changes from those that may occur naturally over time. Without contemporaneous controls, causal inferences cannot be drawn.

Second, although 28 participants initiated the study, only 9 (32%) continued to the 15-month assessment. Because long-term participation was voluntary, those who remained may differ in motivation or engagement. However, baseline comparisons between participants who continued to 15 months and those who participated only in the initial 3-month period showed no significant differences in demographic characteristics, cognitive measures, quality of life scores, or training adherence, as presented in Table S3 in Multimedia Appendix 2. In addition, baseline-to–3-month change scores did not differ between the 2 groups, indicating that early improvement was not greater among those who remained in the study. These findings suggest that systematic baseline differences were limited, although unmeasured factors related to motivation or persistence may still have influenced long-term retention. The small number of long-term participants also restricts the generalizability of the 15-month findings.

Finally, the overall sample size was small, constraining statistical power and limiting the ability to detect subtle effects. Future studies with larger, randomized, and more diverse samples will be essential to determine whether long-term mobile-based cognitive training produces reliable and sustained effects in individuals with MCI.

### Conclusions

This study suggests that a mobile-based MMT app may offer supportive benefits for cognitive function in older adults with MCI. Although methodological limitations constrain definitive interpretation, the observed patterns indicate that mobile-based cognitive training could be a feasible and accessible approach for individuals seeking strategies to maintain cognitive health. Continued evaluation in larger, controlled studies will help determine the extent and durability of these potential effects.

## Appendix

Multimedia Appendix 1 Description of the cognitive exercises included in Cogthera.

Multimedia Appendix 2 Supplementary EQ-5D-5L subdimension results and baseline user comparisons.

## Abbreviations

AD: Alzheimer disease
ADAS-Cog 14: Alzheimer’s Disease Assessment Scale-Cognitive Subscale 14
CDR: Clinical Dementia Rating
CDR-SB: Clinical Dementia Rating-Sum of Boxes
CERAD-NP: Consortium to Establish a Registry for Alzheimer's Disease Neuropsychological Assessment Battery
LASSO: least absolute shrinkage and selection operator
MCI: mild cognitive impairment
MMT: metamemory training

## Data Availability

The datasets generated or analyzed during this study are available from the corresponding author on reasonable request.
