# Supplementary material for: Long-Term Effects of Mobile-Based Metamemory Cognitive Training in Older Adults With Mild Cognitive Impairment: 15-Month Prospective Single-Arm Longitudinal Study
Source: JMIR Aging. 2026 Jan 2;9:e81648. doi: 10.2196/81648 (PMC12811742; doi:10.2196/81648)
Supplement: Multimedia Appendix 2 [file aging_v9i1e81648_app2.docx]

Table S1. Effects of time on EQ-5D-5L subdimension scores.

|  | Baseline  (n=28),  mean (SD) | 3 months  (n=28),  mean (SD) | 15 months  (n=28),  mean (SD) | *F* test  (*df*) | *P* value |
| --- | --- | --- | --- | --- | --- |
| Mobility | 1.29 (0.60) | 1.07 (0.26) | 1.11 (0.33) | 2.59  (2, 39.05) | 0.09 |
| Self-care | 1.04 (0.19) | 1.04 (0.19) | 1.00 (0.00) | 1.39  (2, 41.61) | 0.26 |
| Usual activities | 1.29 (0.53) | 1.25 (0.44) | 1.11 (0.33) | 0.31  (2, 37.30) | 0.74 |
| Pain/discomfort | 1.89 (0.88) | 1.57 (0.69) | 1.33 (0.71) | 2.29  (2, 38.27) | 0.12 |
| Anxiety/depression | 2.11 (0.92) | 1.68 (0.67) | 2.00 (1.22) | 2.15  (2, 39.25) | 0.13 |

Table S2. Longitudinal changes in EQ-5D-5L subdimension scores.

|  | Estimate | SE | *t* test (*df*) | *P* value^a^ |
| --- | --- | --- | --- | --- |
| **Mobility** |  |  |  |  |
| 3 months vs. baseline | -0.21 | 0.10 | -2.23 (35.15) | 0.06 |
| 15 months vs. baseline | -0.17 | 0.15 | -1.15 (42.73) | 0.51 |
| **Self-care** |  |  |  |  |
| 3 months vs. baseline | -0.00 | 0.03 | 0.00 (38.65) | 0.99 |
| 15 months vs. baseline | -0.08 | 0.05 | -1.58 (44.27) | 0.24 |
| **Usual activities** |  |  |  |  |
| 3 months vs. baseline | -0.04 | 0.10 | -0.35 (33.34) | 0.99 |
| 15 months vs. baseline | -0.12 | 0.16 | -0.78 (41.11) | 0.88 |
| **Pain/discomfort** |  |  |  |  |
| 3 months vs. baseline | -0.32 | 0.17 | -1.87 (33.92) | 0.14 |
| 15 months vs. baseline | -0.42 | 0.26 | -1.61 (42.49) | 0.23 |
| **Anxiety/depression** |  |  |  |  |
| 3 months vs. baseline | -0.43 | 0.21 | -2.04 (33.39) | 0.10 |
| 15 months vs. baseline | -0.11 | 0.31 | -0.34 (45.28) | 0.99 |

^a^Bonferroni-adjusted *P* value

Table S3. Comparisons between 3-month users and 15-month users.

|  | 3-month users  (n=19),  mean (SD) | 15-month users  (n=9),  mean (SD) | *t* test (*df*) | *P* value |
| --- | --- | --- | --- | --- |
| Age (years) | 72.6 (6.4) | 73.2 (7.6) | -0.22 (26) | 0.83 |
| Education (years) | 11.8 (5.1) | 10.6 (4.9) | 0.64 (26) | 0.53 |
| CDR-SB^a^ (score) | 2.0 (1.1) | 2.0 (1.0) | -0.00 (26) | 0.99 |
| Compliance (%) | 82.2 (26.4) | 91.8 (15.7) | -1.20 (26) | 0.24 |
| ADAS-cog 14^b^ (total score) | 27.42 (7.73) | 29.78 (9.43) | -0.65 (26) | 0.52 |
| EQ-5D-5L (index score) | 0.81 (0.08) | 0.82 (0.07) | -0.50 (26) | 0.62 |
| Change over 3 months^c^ (score) | 1.32 (3.02) | 0.67 (5.22) | 0.35 (26) | 0.73 |

^a^Clinical Dementia Rating sum of boxes

^b^Alzheimer’s Disease Assessment Scale-cognitive subscale 14. Lower scores represent better performance

^c^Score obtained by subtracting the ADAS-cog 14 score at 3 months from the baseline ADAS-cog 14 score
